# Supplementary material for: Factors affecting template switch recombination associated with restarted DNA replication
Source: eLife. 2019 Jan 22;8:e41697. doi: 10.7554/eLife.41697 (PMC6358216; doi:10.7554/eLife.41697)
Supplement: Supplementary file 1. [file elife-41697-supp1.docx]

**SUPPLEMENTARY FILE 1**

**Direct repeat recombinant frequencies**

| Relevant Genotype  and  Strain no. | *RTS1* presence and orientation ^a^ | Position of direct repeat relative to site of *RTS1* insertion^b^ | Number of colonies analysed | Ade^+^ His^+^  recombinant  frequency (x 10^-4^)^c^ | | Ade^+^ His^-^  recombinant  frequency (x 10^-4^)^c^ | |
| --- | --- | --- | --- | --- | --- | --- | --- |
|  |  |  |  | Mean | *P* value^d^ | Mean | *P* value^d^ |
| wild-type MCW429 | - | 0 kb | 20 | 1.10  (+/-0.37) | - | 2.63  (+/-0.51) | - |
| wild-type MCW4712 | IO | 0 kb  flanking | 96 | 1.38  (+/-0.51) | 0.005^e^ | 3.57  (+/-0.99) | <.001^e^ |
| wild-type MCW4713 | AO | 0 kb  flanking | 102 | 135.4  (+/-38.8) | <0.0001^f^ | 107.9  (+/-45.7) | <0.0001^f^ |
| wild-type MCW7131 | IO | 0.2 kb down-stream | 47 | 1.25  (+/-0.70) | 0.837^e^ | 5.05  (+/-2.25) | <.0001^e^ |
| wild-type MCW7133 | AO | 0.2 kb down-stream | 45 | 157.4  (±66.0) | <0.0001^g^ | 682.4  (±286.5) | <0.0001^g^ |
| *ori-1253*∆ MCW7414 | IO | 0.2 kb down-stream | 38 | 1.46  (±0.47) | 0.002^g^ | 4.52  (±1.63) | 0.331^g^ |
| *ori-1253*∆ MCW7416 | AO | 0.2 kb down-stream | 40 | 280.28  (±130.43) | <0.0001^h^ | 465.35  (±166.99) | <0.0001^h^ |
| wild-type MCW7229 | - | 12.4 kb down-stream | 17 | 0.77  (+/-0.29) | - | 1.14  (+/-1.07) | - |
| wild-type MCW7257 | IO | 12.4 kb down-stream | 23 | 0.84  (±0.49) | 0.837^i^ | 2.01  (±0.77) | 0.001^i^ |
| wild-type MCW7259 | AO | 12.4 kb down-stream | 104 | 4.95  (±2.54) | <0.0001^j^ | 84.85  (±27.71) | <0.0001^j^ |
| *ori-1253*∆ MCW7293 | IO | 12.4 kb down-stream | 22 | 1.83  (±0.84) | <0.0001^j^ | 4.27  (±1.50) | <0.0001^j^ |
| *ori-1253*∆ MCW7295 | AO | 12.4 kb down-stream | 26 | 103.45  (±39.48) | <0.0001^k^ | 1006.41  (±429.81) | <0.0001^k^ |
| wild-type MCW7429 | - | 35 kb down-stream | 17 | 4.25  (+/-2.41) | - | 0.86  (+/-0.69) | - |
| wild-type MCW7565 | IO | 35 kb down-stream | 13 | 3.75  (±2.21) | 0.722^l^ | 0.69  (±0.54) | 0.414^l^ |
| wild-type MCW7567 | AO | 35 kb down-stream | 17 | 61.39  (±30.77) | <0.0001^m^ | 39.98  (±25.13) | <0.0001^m^ |
| wild-type MCW7430 | - | 75 kb down-stream | 30 | 1.09  (+/-0.46) | - | 2.28  (+/-0.63) | - |
| wild-type MCW7614 | IO | 75 kb down-stream | 18 | 1.25  (±0.55) | 0.431^n^ | 2.56  (±0.93) | 0.406^n^ |
| wild-type MCW7616 | AO | 75 kb down-stream | 15 | 1.76  (±0.99) | 0.169^o^ | 3.04  (±1.00) | 0.181^o^ |
| *ori-1253*∆ MCW7987 | - | 75 kb down-stream | 16 | 1.19  (±0.49) | 0.580^n^ | 2.97  (±1.66) | 0.344^n^ |
| *ori-1253*∆ MCW7620 | AO | 75 kb down-stream | 15 | 2.35  (±0.82) | 0.141^p^ | 16.34  (±8.85) | <0.0001^p^ |
| wild-type MCW7297 | - | 140 kb down-stream | 9 | 0.87  (±0.22) | - | 2.09  (±0.75) | - |
| wild-type MCW7326 | IO | 140 kb down-stream | 13 | 1.05  (±0.61) | 0.526^q^ | 2.58  (±1.22) | 0.443^q^ |
| wild-type MCW7328 | AO | 140 kb down-stream | 12 | 1.19  (±0.62) | 0.211^r^ | 2.69  (±1.01) | 0.550^r^ |
| *pfh1-m21* MCW4940 | IO | Flanking | 9 | 1.41  (+/-0.39) | 0.909^f^ | 3.57  (+/-0.62) | 0.655^f^ |
| *pfh1-m21* MCW4942 | AO | Flanking | 28 | 164.4  (+/-57.7) | 0.009^s^ | 1778.9  (+/-359.3) | <0.0001^s^ |
| *pfh1-mt** MCW4954 | IO | Flanking | 10 | 2.09  (+/-0.49) | <0.0001^f^ | 14.4  (+/-4.65) | <0.0001^f^ |
| *pfh1-mt** MCW4956 | AO | Flanking | 20 | 176.4  (+/-44.5) | <0.0001^s^ | 1610.5  (+/-257.0) | <0.0001^s^ |
| *pfh1-m21* MCW7599 | IO | 0.2 kb down-stream | 20 | 1.51  (+/-0.41) | 0.004^g^ | 4.00  (+/-1.10) | 0.059^g^ |
| *pfh1-m21* MCW7601 | AO | 0.2 kb down-stream | 19 | 39.31  (±25.08) | <0.0001^h^ | 779.2  (±168.2) | 0.012^h^ |
| *pfh1-m21 ori-1253*∆ MCW7598 | IO | 0.2 kb down-stream | 20 | 2.82  (+/-1.79) | <0.0001^v^ | 8.00  (+/-3.70) | <0.0001^v^ |
| *pfh1-m21 ori-1253*∆ MCW7600 | AO | 0.2 kb down-stream | 12 | 134.9  (+/-71.6) | <0.0001^w^ | 1648.9  (+/-288.8) | <0.0001^w^ |
| *pfh1-mt** MCW7603 | IO | 0.2 kb down-stream | 18 | 2.58  (+/-0.88) | <0.0001^g^ | 7.30  (+/-2.20) | <0.0001^g^ |
| *pfh1-mt** MCW7605 | AO | 0.2 kb down-stream | 20 | 4.85  (+/-2.19) | <0.0001^h^ | 246.7  (+/-141.4) | <0.0001^h^ |
| *pfh1-mt* ori-1253*∆ MCW7602 | IO | 0.2 kb down-stream | 21 | 4.34  (±2.09) | <0.0001^v^ | 10.80  (±4.63) | <0.0001^v^ |
| *pfh1-mt* ori-1253*∆ MCW7604 | AO | 0.2 kb down-stream | 20 | 16.69  (±10.53) | <0.0001^w^ | 446.24  (±117.77) | 0.471^w^ |
| *pfh1-m21* MCW7421 | IO | 12.4 kb down-stream | 18 | 2.14  (+/-1.23) | <0.0001^j^ | 3.20  (+/-1.28) | 0.001^j^ |
| *pfh1-m21* MCW7422 | AO | 12.4 kb down-stream | 17 | 2.05  (+/-0.85) | <0.0001^t^ | 10.4  (+/-3.07) | <0.0001^t^ |
| *pfh1-m21 ori-1253*∆ MCW7423 | IO | 12.4 kb down-stream | 19 | 1.60  (+/-0.40) | 0.548^x^ | 5.03  (+/-1.41) | 0.067^x^ |
| *pfh1-m21 ori-1253*∆ MCW7424 | AO | 12.4 kb down-stream | 20 | 9.15  (+/-2.38) | <0.0001^y^ | 121.7  (+/-55.3) | <0.0001^y^ |
| *pfh1-mt** MCW7425 | IO | 12.4 kb down-stream | 17 | 1.24  (+/-0.49) | 0.005^j^ | 2.38  (+/-1.20) | 0.315^j^ |
| *pfh1-mt** MCW7426 | AO | 12.4 kb down-stream | 18 | 1.49  (+/-0.64) | <0.0001^t^ | 5.05  (+/-1.55) | <0.0001^t^ |
| *pfh1-mt* ori-1253*∆ MCW7427 | IO | 12.4 kb down-stream | 15 | 1.71  (+/-0.52) | 0.757^x^ | 4.17  (+/-1.30) | 0.829^x^ |
| *pfh1-mt* ori-1253*∆ MCW7428 | AO | 12.4 kb down-stream | 17 | 4.04  (+/-1.66) | <0.0001^y^ | 16.8  (+/-5.33) | <0.0001^y^ |
| *tRNA* co-directional MCW7434 | - | 12.4 kb down-stream | 16 | 0.75  (+/-0.27) | 0.692^i^ | 1.07  (+/-0.48) | 0.113^i^ |
| *tRNA* co-directional MCW7521 | AO | 12.4 kb down-stream | 17 | 4.19  (±1.31) | 0.345^t^ | 70.1  (±25.5) | 0.015^t^ |
| *tRNA*  head-on MCW7433 | - | 12.4 kb down-stream | 16 | 1.26  (±0.46) | 0.005^i^ | 1.60  (±0.62) | 0.005^i^ |
| *tRNA*  head-on MCW7517 | AO | 12.4 kb down-stream | 21 | 5.11  (±2.71) | 0.924^t^ | 200.5  (±68.1) | <0.0001^t^ |
| *pfh1-m21*  *tRNA*  head-on MCW9381 | - | 12.4 kb down-stream | 23 | 10.96  (±5.13) | <0.0001^z^ | 38.55  (±17.22) | <0.0001^z^ |
| *pfh1-m21*  *tRNA*  head-on MCW9360 | AO | 12.4 kb down-stream | 25 | 3.45  (±1.46) | 0.018^aa^ | 19.24  (±8.50) | <0.0001^aa^ |
| *pfh1-mt**  *tRNA*  head-on MCW9383 | - | 12.4 kb down-stream | 22 | 24.26  (±12.48) | <0.0001^z^ | 66.17  (±28.44) | <0.0001^z^ |
| *pfh1-mt**  *tRNA*  head-on MCW9361 | AO | 12.4 kb down-stream | 23 | 2.96  (±1.61) | 0.0034^aa^ | 15.48  (±7.60) | <0.0001^aa^ |
| *rqh1*∆ MCW1443 | IO | Flanking | 10 | 2.62  (+/-0.92) | <0.0001^f^ | 14.7  (+/-3.29) | <0.0001^f^ |
| *rqh1*∆ MCW1447 | AO | Flanking | 11 | 357.6  (+/-209.5) | <0.0001^s^ | 2167.3  (+/-848.9) | <0.0001^s^ |
| *rqh1*∆ MCW8201 | AO | 12.4 kb down-stream | 19 | 11.1  (+/-3.98) | <0.0001^t^ | 250.8  (+/-91.0) | <0.0001^t^ |
| *srs2*∆  FO1748^ab^ | IO | Flanking | 15 | 12.9  (+/-6.00) | <0.0001^f^ | 17.0  (+/-9.20) | <0.0001^f^ |
| *srs2*∆  FO1750^ab^ | AO | Flanking | 16 | 1048.2  (+/-279.8) | <0.0001^s^ | 548.2  (+/-148.2) | <0.0001^s^ |
| *srs2*∆ MCW8200 | AO | 12.4 kb down-stream | 20 | 103.3  (+/-39.9) | <0.0001^t^ | 462.3  (+/-144.7) | <0.0001^t^ |
| *fbh1*∆ FO1814^ab^ | IO | Flanking | 22 | 1.62  (+/-0.54) | 0.052^f^ | 3.35  (+/-0.82) | 0.316^f^ |
| *fbh1*∆ FO1816 | AO | Flanking | 15 | 916.7  (+/-455.5) | <0.0001^s^ | 2165.5  (+/-662.2) | <0.0001^s^ |
| *fbh1*∆ MCW8227 | AO | 12.4 kb down-stream | 15 | 63.9  (+/-33.5) | <0.0001^t^ | 801.6  (+/-230.5) | <0.0001^t^ |
| *fml1∆* MCW3059 | IO | Flanking | 16 | 0.53  (+/-0.29) | <0.0001^f^ | 3.41  (+/-1.29) | 0.649^f^ |
| *fml1∆* MCW3061 | AO | Flanking | 22 | 17.2  (+/-8.9) | <0.0001^s^ | 108.3  (+/-53.5) | 0.974^s^ |
| *fml1∆* MCW8193 | AO | 12.4 kb down-stream | 17 | 4.5  (+/-2.8) | 0.501^t^ | 94.3  (+/-24.6) | 0.164^t^ |
| *mus81*∆ MCW1451 | IO | Flanking | 21 | 1.29  (+/-0.78) | 0.087^f^ | 3.00  (+/-1.57) | 0.01^f^ |
| *mus81*∆ MCW1452 | AO | Flanking | 23 | 132.5  (+/-42.3) | 0.464^s^ | 149.2  (+/-77.8) | 0.002^s^ |
| *mus81*∆ MCW8195 | AO | 12.4 kb down-stream | 16 | 4.95  (+/-1.20) | 0.342^t^ | 55.1  (+/-14.4) | <0.0001^t^ |
| *pcf2*∆ MCW6972 | IO | Flanking | 16 | 2.19  (+/-0.97) | <0.005^f^ | 3.97  (+/-1.14) | 0.194^f^ |
| *pcf2*∆ MCW7213 | AO | Flanking | 33 | 36.1  (+/-12.5) | <0.0001^s^ | 69.3  (+/-24.6) | <0.0001^s^ |
| *pcf2*∆ MCW8359 | AO | 12.4 kb down-stream | 14 | 1.92  (+/-1.04) | <0.0001^t^ | 20.7  (+/-12.2) | <0.0001^t^ |
| *pcf3*∆ MCW7147 | IO | Flanking | 18 | 1.99  (+/-0.53) | <0.001^f^ | 4.48  (+/-1.28) | 0.009^f^ |
| *pcf3*∆ MCW7149 | AO | Flanking | 15 | 45.9  (+/-17.6) | <0.0001^s^ | 74.0  (+/-27.0) | 0.001^s^ |
| *pcf3*∆ MCW8360 | AO | 12.4 kb down-stream | 13 | 2.44  (+/-1.47) | <0.0001^t^ | 24.2  (+/-8.2) | <0.0001^t^ |

^a^ *RTS1* is integrated at the *ade6* locus either between a direct repeat of *ade6^-^* alleles or replacing *ade6*. In strains without *RTS1*, the *ade6* gene is deleted from its normal locus.

^b^ The different positions of the *ade6^-^* direct repeat recombination reporter are shown in Figure 1A.

^c^ The values in parentheses are the standard deviations about the mean.

^d^ *P* values are calculated by a two-tailed Mann-Whitney U test comparing the mean values as indicated.

^e^ Compared to the equivalent mean recombinant frequency of MCW429.

^f^ Compared to the equivalent mean recombinant frequency of MCW4712.

^g^ Compared to the equivalent mean recombinant frequency of MCW7131.

^h^ Compared to the equivalent mean recombinant frequency of MCW7133.

^i^ Compared to the equivalent mean recombinant frequency of MCW7229.

^j^ Compared to the equivalent mean recombinant frequency of MCW7257.

^k^ Compared to the equivalent mean recombinant frequency of MCW7293.

^l^ Compared to the equivalent mean recombinant frequency of MCW7429.

^m^ Compared to the equivalent mean recombinant frequency of MCW7565.

^n^ Compared to the equivalent mean recombinant frequency of MCW7430.

^o^ Compared to the equivalent mean recombinant frequency of MCW7614.

^p^ Compared to the equivalent mean recombinant frequency of MCW7616.

^q^ Compared to the equivalent mean recombinant frequency of MCW7297.

^r^ Compared to the equivalent mean recombinant frequency of MCW7326.

^s^ Compared to the equivalent mean recombinant frequency of MCW4713.

^t^ Compared to the equivalent mean recombinant frequency of MCW7259.

^u^ Compared to the equivalent mean recombinant frequency of MCW7326.

^v^ Compared to the equivalent mean recombinant frequency of MCW7414.

^w^ Compared to the equivalent mean recombinant frequency of MCW7416.

^x^ Compared to the equivalent mean recombinant frequency of MCW7293.

^y^ Compared to the equivalent mean recombinant frequency of MCW7295.

^z^ Compared to the equivalent mean recombinant frequency of MCW7433.

^aa^ Compared to the equivalent mean recombinant frequency of MCW7517.

^ab^ Data from (Lorenz et al., 2009).
