# Supplementary material for: Factors affecting template switch recombination associated with restarted DNA replication
Source: eLife. 2019 Jan 22;8:e41697. doi: 10.7554/eLife.41697 (PMC6358216; doi:10.7554/eLife.41697)
Supplement: Supplementary file 2. [file elife-41697-supp2.docx]

**SUPPLEMENTARY FILE 2**

***Schizosccharomyces pombe* strains** (in order of appearance)

| **Strain** | **Relevant genotype** | **Source** |
| --- | --- | --- |
| MCW429 | *h^+^ ade6-M375 int*::*pUC8/his3^+^/ade6-L469 ura4-D18 his3-D1 leu1-32 arg3-D4* | (Ahn et al., 2005) |
| MCW4712 | *h^+^ ade6-M375 int*::*pUC8/his3^+^/RTS1-IO/ade6-L469 ura4-D18 his3-D1 leu1-32 arg3-D4* | (Ahn et al., 2005) |
| MCW4713 | *h^+^ ade6-M375 int*::*pUC8/his3^+^/RTS1-AO/ade6-L469 ura4-D18 his3-D1 leu1-32 arg3-D4* | (Ahn et al., 2005) |
| MCW7131 | *h^+^ ade6-M375 int*::*pUC8/his3^+^/ade6-L469/RTS1-IO/hphMX4 ura4-D18 his3-D1 leu1-32 arg3-D4* | (Nguyen et al., 2015) |
| MCW7133 | *h^+^ ade6-M375 int*::*pUC8/his3^+^/ade6-L469/RTS1-AO/hphMX4 ura4-D18 his3-D1 leu1-32 arg3-D4* | (Nguyen et al., 2015) |
| MCW7414 | *h^+^ ori1253∆*::*natMX4 ade6-M375 int*::*pUC8/his3^+^/ade6-L469/RTS1-IO/hphMX4 ura4-D18 his3-D1 leu1-32 arg3-D4* | This study |
| MCW7416 | *h^+^ ori1253∆*::*natMX4 ade6-M375 int*::*pUC8/his3^+^/ade6-L469/RTS1-AO/hphMX4 ura4-D18 his3-D1 leu1-32 arg3-D4* | This study |
| MCW7229 | *h^+^*(12.4 kb from *ade6*)int::*ade6-M375 int*::*pUC8/his3^+^/ade6-L469/kanMX6 ade6-D1 ura4-D18 his3-D1 leu1-32 arg3-D4* | This study |
| MCW7257 | *h^+^ ade6*∆::*RTS1-IO-hphMX4* (12.4 kb from *ade6*)int::*ade6-M375 int*::*pUC8/his3^+^/ade6-L469/kanMX6 ura4-D18 his3-D1 leu1-32 arg3-D4* | (Nguyen et al., 2015) |
| MCW7259 | *h^+^ ade6*∆::*RTS1-AO-hphMX4* (12.4 kb from *ade6*)int::*ade6-M375 int*::*pUC8/his3^+^/ade6-L469/kanMX6 ura4-D18 his3-D1 leu1-32 arg3-D4* | (Nguyen et al., 2015) |
| MCW7293 | *h^+^ ori1253∆*::*natMX4 ade6*∆::*RTS1-IO-hphMX4* (12.4 kb from *ade6*)int::*ade6-M375 int*::*pUC8/his3^+^/ade6-L469/kanMX6 ura4-D18 his3-D1 leu1-32 arg3-D4* | (Nguyen et al., 2015) |
| MCW7295 | *h^+^ ori1253∆*::*natMX4 ade6*∆::*RTS1-AO-hphMX4* (12.4 kb from *ade6*)int::*ade6-M375 int*::*pUC8/his3^+^/ade6-L469/kanMX6 ura4-D18 his3-D1 leu1-32 arg3-D4* | (Nguyen et al., 2015) |
| MCW7429 | *h^+^*(35 kb from *ade6*)int::*ade6-M375 int*::*pUC8/his3^+^/ade6-L469/kanMX6 ade6-D1 ura4-D18 his3-D1 leu1-32 arg3-D4* | This study |
| MCW7565 | *h^+^ ade6*∆::*RTS1-IO-hphMX4* (35 kb from *ade6*)int::*ade6-M375 int*::*pUC8/his3^+^/ade6-L469/kanMX6 ura4-D18 his3-D1 leu1-32 arg3-D4* | This study |
| MCW7567 | *h^+^ ade6*∆::*RTS1-AO-hphMX4* (35 kb from *ade6*)int::*ade6-M375 int*::*pUC8/his3^+^/ade6-L469/kanMX6 ura4-D18 his3-D1 leu1-32 arg3-D4* | This study |
| MCW7430 | *h^+^*(75 kb from *ade6*)int::*ade6-M375 int*::*pUC8/his3^+^/ade6-L469/kanMX6 ade6-D1 ura4-D18 his3-D1 leu1-32 arg3-D4* | This study |
| MCW7614 | *h^+^ ade6*∆::*RTS1-IO-hphMX4* (75 kb from *ade6*)int::*ade6-M375 int*::*pUC8/his3^+^/ade6-L469/kanMX6 ura4-D18 his3-D1 leu1-32 arg3-D4* | This study |
| MCW7616 | *h^+^ ade6*∆::*RTS1-AO-hphMX4* (75 kb from *ade6*)int::*ade6-M375 int*::*pUC8/his3^+^/ade6-L469/kanMX6 ura4-D18 his3-D1 leu1-32 arg3-D4* | This study |
| MCW7987 | *h^+^ ori1253∆*::*arg3*^+^ (75 kb from *ade6*)int::*ade6-M375 int*::*pUC8/his3^+^/ade6-L469/kanMX6 ade6-D1 ura4-D18 his3-D1 leu1-32 arg3-D4* | This study |
| MCW7620 | *h^+^ ori1253∆*::*natMX4 ade6*∆::*RTS1-AO-hphMX4* (75 kb from *ade6*)int::*ade6-M375 int*::*pUC8/his3^+^/ade6-L469/kanMX6 ura4-D18 his3-D1 leu1-32 arg3-D4* | This study |
| MCW7297 | *h^+^*(140 kb from *ade6*)int::*ade6-M375 int*::*pUC8/his3^+^/ade6-L469/kanMX6 ade6-D1 ura4-D18 his3-D1 leu1-32 arg3-D4* | This study |
| MCW7326 | *h^+^ ade6*∆::*RTS1-IO-hphMX4* (140 kb from *ade6*)int::*ade6-M375 int*::*pUC8/his3^+^/ade6-L469/kanMX6 ura4-D18 his3-D1 leu1-32 arg3-D4* | This study |
| MCW7328 | *h^+^ ade6*∆::*RTS1-AO-hphMX4* (140 kb from *ade6*)int::*ade6-M375 int*::*pUC8/his3^+^/ade6-L469/kanMX6 ura4-D18 his3-D1 leu1-32 arg3-D4* | This study |
| MCW4940 | *h^+^ pfh1∆*::*kanMX6 leu1-32*::*pJK148/leu1^+^/pfh1-m21 ade6-M375 int*::*pUC8/his3^+^/RTS1-IO/ade6-L469 ura4-D18 his3-D1 arg3-D4* | This study |
| MCW4942 | *h^+^ pfh1∆*::*kanMX6 leu1-32*::*pJK148/leu1^+^/pfh1-m21 ade6-M375 int*::*pUC8/his3^+^/RTS1-AO/ade6-L469 ura4-D18 his3-D1 arg3-D4* | This study |
| MCW4954 | *h^+^ pfh1∆*::*kanMX6 leu1-32*::*pJK148/leu1^+^/pfh1-mt* ade6-M375 int*::*pUC8/his3^+^/RTS1-IO/ade6-L469 ura4-D18 his3-D1 arg3-D4* | This study |
| MCW4956 | *h^+^ pfh1∆*::*kanMX6 leu1-32*::*pJK148/leu1^+^/pfh1-mt* ade6-M375 int*::*pUC8/his3^+^/RTS1-AO/ade6-L469 ura4-D18 his3-D1 arg3-D4* | (Steinacher et al., 2012) |
| MCW7599 | *h^-^ pfh1∆*::*arg3^+^ leu1-32*::*pJK148/leu1^+^/pfh1-m21 ade6-M375 int*::*pUC8/his3^+^/ade6-L469/RTS1-IO/hphMX4 ura4-D18 his3-D1 arg3-D4* | This study |
| MCW7601 | *h^+^ pfh1∆*::*arg3^+^ leu1-32*::*pJK148/leu1^+^/pfh1-m21 ade6-M375 int*::*pUC8/his3^+^/ade6-L469/RTS1-AO/hphMX4 ura4-D18 his3-D1 arg3-D4* | This study |
| MCW7603 | *h^+^ pfh1∆*::*arg3^+^ leu1-32*::*pJK148/leu1^+^/pfh1-mt* ade6-M375 int*::*pUC8/his3^+^/ade6-L469/RTS1-IO/hphMX4 ura4-D18 his3-D1 arg3-D4* | This study |
| MCW7605 | *h^+^ pfh1∆*::*arg3^+^ leu1-32*::*pJK148/leu1^+^/pfh1-mt* ade6-M375 int*::*pUC8/his3^+^/ade6-L469/RTS1-AO/hphMX4 ura4-D18 his3-D1 arg3-D4* | This study |
| MCW7421 | *h^+^ pfh1∆*::*arg3^+^ leu1-32*::*pJK148/leu1^+^/pfh1-m21 ade6*∆::*RTS1-IO-hphMX4* (12.4 kb from *ade6*)int::*ade6-M375 int*::*pUC8/his3^+^/ade6-L469/kanMX6 ura4-D18 his3-D1 leu1-32 arg3-D4* | This study |
| MCW7422 | *h^+^ pfh1∆*::*arg3^+^ leu1-32*::*pJK148/leu1^+^/pfh1-m21 ade6*∆::*RTS1-AO-hphMX4* (12.4 kb from *ade6*)int::*ade6-M375 int*::*pUC8/his3^+^/ade6-L469/kanMX6 ura4-D18 his3-D1 leu1-32 arg3-D4* | This study |
| MCW7425 | *h^+^ pfh1∆*::*arg3^+^ leu1-32*::*pJK148/leu1^+^/pfh1-mt* ade6*∆::*RTS1-IO-hphMX4* (12.4 kb from *ade6*)int::*ade6-M375 int*::*pUC8/his3^+^/ade6-L469/kanMX6 ura4-D18 his3-D1 leu1-32 arg3-D4* | This study |
| MCW7426 | *h^+^ pfh1∆*::*arg3^+^ leu1-32*::*pJK148/leu1^+^/pfh1-mt* ade6*∆::*RTS1-AO-hphMX4* (12.4 kb from *ade6*)int::*ade6-M375 int*::*pUC8/his3^+^/ade6-L469/kanMX6 ura4-D18 his3-D1 leu1-32 arg3-D4* | This study |
| MCW7223 | *h^-^ ade6*∆::*RTS1-AO-hphMX4 ura4-D18 his3-D1 leu1-32 arg3-D4* | (Nguyen et al., 2015) |
| MCW8587 | *h^+^ pfh1∆*::*arg3^+^ leu1-32*::*pJK148/leu1^+^/pfh1-mt* ade6*∆::*RTS1-IO-hphMX4 ura4-D18 his3-D1 leu1-32 arg3-D4* | This study |
| MCW8605 | *h^+^ pfh1∆*::*arg3^+^ leu1-32*::*pJK148/leu1^+^/pfh1-mt* exo1*∆::*kanMX6 ade6*∆::*RTS1-IO-hphMX4 ura4-D18 his3-D1 leu1-32 arg3-D4* | This study |
| MCW7598 | *h^+^ ori1253∆*::*natMX4 pfh1∆*::*arg3^+^ leu1-32*::*pJK148/leu1^+^/pfh1-m21 ade6-M375 int*::*pUC8/his3^+^/ade6-L469/RTS1-IO/hphMX4 ura4-D18 his3-D1 arg3-D4* | This study |
| MCW7600 | *h^+^ ori1253∆*::*natMX4 pfh1∆*::*arg3^+^ leu1-32*::*pJK148/leu1^+^/pfh1-m21 ade6-M375 int*::*pUC8/his3^+^/ade6-L469/RTS1-AO/hphMX4 ura4-D18 his3-D1 arg3-D4* | This study |
| MCW7602 | *h^+^ ori1253∆*::*natMX4 pfh1∆*::*arg3^+^ leu1-32*::*pJK148/leu1^+^/pfh1-mt* ade6-M375 int*::*pUC8/his3^+^/ade6-L469/RTS1-IO/hphMX4 ura4-D18 his3-D1 arg3-D4* | This study |
| MCW7604 | *h^+^ ori1253∆*::*natMX4 pfh1∆*::*arg3^+^ leu1-32*::*pJK148/leu1^+^/pfh1-mt* ade6-M375 int*::*pUC8/his3^+^/ade6-L469/RTS1-AO/hphMX4 ura4-D18 his3-D1 arg3-D4* | This study |
| MCW7423 | *h^+^ ori1253∆*::*natMX4 pfh1∆*::*arg3^+^ leu1-32*::*pJK148/leu1^+^/pfh1-m21 ade6*∆::*RTS1-IO-hphMX4* (12.4 kb from *ade6*)int::*ade6-M375 int*::*pUC8/his3^+^/ade6-L469/kanMX6 ura4-D18 his3-D1 leu1-32 arg3-D4* | This study |
| MCW7424 | *h^+^ ori1253∆*::*natMX4 pfh1∆*::*arg3^+^ leu1-32*::*pJK148/leu1^+^/pfh1-m21 ade6*∆::*RTS1-AO-hphMX4* (12.4 kb from *ade6*)int::*ade6-M375 int*::*pUC8/his3^+^/ade6-L469/kanMX6 ura4-D18 his3-D1 leu1-32 arg3-D4* | This study |
| MCW7427 | *h^-^ ori1253∆*::*natMX4 pfh1∆*::*arg3^+^ leu1-32*::*pJK148/leu1^+^/pfh1-mt* ade6*∆::*RTS1-IO-hphMX4* (12.4 kb from *ade6*)int::*ade6-M375 int*::*pUC8/his3^+^/ade6-L469/kanMX6 ura4-D18 his3-D1 leu1-32 arg3-D4* | This study |
| MCW7428 | *h^-^ ori1253∆*::*natMX4 pfh1∆*::*arg3^+^ leu1-32*::*pJK148/leu1^+^/pfh1-mt* ade6*∆::*RTS1-AO-hphMX4* (12.4 kb from *ade6*)int::*ade6-M375 int*::*pUC8/his3^+^/ade6-L469/kanMX6 ura4-D18 his3-D1 leu1-32 arg3-D4* | This study |
| MCW7434 | *h^+^*(12.4 kb from *ade6*)int::*ade6-M375 int*::*pUC8/his3^+^/tRNA^GLU08^*CD*)ade6-L469/kanMX6 ade6-D1 ura4-D18 his3-D1 leu1-32 arg3-D4* | This study |
| MCW7433 | *h^+^*(12.4 kb from *ade6*)int::*ade6-M375 int*::*pUC8/his3^+^/tRNA^GLU08^*HO*)ade6-L469/kanMX6 ade6-D1 ura4-D18 his3-D1 leu1-32 arg3-D4* | This study |
| MCW7521 | *h^+^ ade6*∆::*RTS1-AO-hphMX4* (12.4 kb from *ade6*)int::*ade6-M375 int*::*pUC8/his3^+^/tRNA^GLU08^*CD*)ade6-L469/kanMX6 ura4-D18 his3-D1 leu1-32 arg3-D4* | This study |
| MCW7517 | *h^+^ ade6*∆::*RTS1-AO-hphMX4* (12.4 kb from *ade6*)int::*ade6-M375 int*::*pUC8/his3^+^/tRNA^GLU08^*HO*)ade6-L469/kanMX6 ura4-D18 his3-D1 leu1-32 arg3-D4* | This study |
| MCW9381 | *h^+^ pfh1∆*::*arg3^+^ leu1-32*::*pJK148/leu1^+^/pfh1-*m21 (12.4 kb from *ade6*)int::*ade6-M375 int*::*pUC8/his3^+^/tRNA^GLU08^*HO*)ade6-L469/kanMX6 ade6-D1 ura4-D18 his3-D1 leu1-32 arg3-D4* | This study |
| MCW9383 | *h^+^ pfh1∆*::*arg3^+^ leu1-32*::*pJK148/leu1^+^/pfh1-mt** (12.4 kb from *ade6*)int::*ade6-M375 int*::*pUC8/his3^+^/tRNA^GLU08^*HO*)ade6-L469/kanMX6 ade6-D1 ura4-D18 his3-D1 leu1-32 arg3-D4* | This study |
| MCW9360 | *h^+^ pfh1∆*::*arg3^+^ leu1-32*::*pJK148/leu1^+^/pfh1-m21 ade6*∆::*RTS1-AO-hphMX4* (12.4 kb from *ade6*)int::*ade6-M375 int*::*pUC8/his3^+^/tRNA^GLU08^*HO*)ade6-L469/kanMX6 ura4-D18 his3-D1 leu1-32 arg3-D4* | This study |
| MCW9361 | *h^+^ pfh1∆*::*arg3^+^ leu1-32*::*pJK148/leu1^+^/pfh1-mt* ade6*∆::*RTS1-AO-hphMX4* (12.4 kb from *ade6*)int::*ade6-M375 int*::*pUC8/his3^+^/tRNA^GLU08^*HO*)ade6-L469/kanMX6 ura4-D18 his3-D1 leu1-32 arg3-D4* | This study |
| MCW1443 | *h^-^ rqh1*∆::*kanMX6 ade6-M375 int*::*pUC8/his3^+^/RTS1-IO/ade6-L469 ura4-D18 his3-D1 leu1-32 arg3-D4* | (Ahn et al., 2005) |
| MCW1447 | *h^-^ rqh1*∆::*kanMX6 ade6-M375 int*::*pUC8/his3^+^/RTS1-AO/ade6-L469 ura4-D18 his3-D1 leu1-32 arg3-D4* | (Ahn et al., 2005) |
| MCW8201 | *h^+^ rqh1*∆::*ura4^+^ ade6*∆::*RTS1-AO-hphMX4* (12.4 kb from *ade6*)int::*ade6-M375 int*::*pUC8/his3^+^/ade6-L469/kanMX6 ura4-D18 his3-D1 leu1-32 arg3-D4* | This study |
| FO1748 | *h^+^ srs2*∆::*ura4^+^ ade6-M375 int*::*pUC8/his3^+^/RTS1-IO/ade6-L469 ura4-D18 his3-D1 leu1-32 arg3-D4* | (Lorenz et al., 2009) |
| FO1750 | *h^+^ srs2*∆::*ura4^+^ ade6-M375 int*::*pUC8/his3^+^/RTS1-AO/ade6-L469 ura4-D18 his3-D1 leu1-32 arg3-D4* | (Lorenz et al., 2009) |
| MCW8200 | *h^-^ srs2*∆::*ura4^+^ ade6*∆::*RTS1-AO-hphMX4* (12.4 kb from *ade6*)int::*ade6-M375 int*::*pUC8/his3^+^/ade6-L469/kanMX6 ura4-D18 his3-D1 leu1-32 arg3-D4* | This study |
| FO1814 | *h^+^ fbh1*∆::*kanMX6 ade6-M375 int*::*pUC8/his3^+^/RTS1-IO/ade6-L469 ura4-D18 his3-D1 leu1-32 arg3-D4* | (Lorenz et al., 2009) |
| FO1816 | *h^+^ fbh1*∆::*kanMX6 ade6-M375 int*::*pUC8/his3^+^/RTS1-AO/ade6-L469 ura4-D18 his3-D1 leu1-32 arg3-D4* | (Lorenz et al., 2009) |
| MCW8227 | *h^-^ fbh1*∆::*arg3^+^ ade6*∆::*RTS1-AO-hphMX4* (12.4 kb from *ade6*)int::*ade6-M375 int*::*pUC8/his3^+^/ade6-L469/kanMX6 ura4-D18 his3-D1 leu1-32 arg3-D4* | This study |
| MCW3059 | *h^+^ fml1*∆*::natMX4 ade6-M375 int*::*pUC8/his3^+^/RTS1-IO/ade6-L469 ura4-D18 his3-D1 leu1-32 arg3-D4* | (Sun et al., 2008) |
| MCW3061 | *h^+^ fml1*∆*::natMX4 ade6-M375 int*::*pUC8/his3^+^/RTS1-AO/ade6-L469 ura4-D18 his3-D1 leu1-32 arg3-D4* | (Sun et al., 2008) |
| MCW8193 | *h^+^ fml1*∆*::natMX4 ade6*∆::*RTS1-AO-hphMX4* (12.4 kb from *ade6*)int::*ade6-M375 int*::*pUC8/his3^+^/ade6-L469/kanMX6 ura4-D18 his3-D1 leu1-32 arg3-D4* | This study |
| MCW1451 | *h^?^ mus81*∆*::kanMX6 ade6-M375 int*::*pUC8/his3^+^/RTS1-IO/ade6-L469 ura4-D18 his3-D1 leu1-32 arg3-D4* | Lab strain |
| MCW1452 | *h^?^ mus81*∆*::kanMX6 ade6-M375 int*::*pUC8/his3^+^/RTS1-AO/ade6-L469 ura4-D18 his3-D1 leu1-32 arg3-D4* | Lab strain |
| MCW8195 | *h^+^ mus81*∆*::arg3^+^ ade6*∆::*RTS1-AO-hphMX4* (12.4 kb from *ade6*)int::*ade6-M375 int*::*pUC8/his3^+^/ade6-L469/kanMX6 ura4-D18 his3-D1 leu1-32 arg3-D4* | This study |
| MCW6972 | *h^+^ pcf2*∆*::kanMX6 ade6-M375 int*::*pUC8/his3^+^/RTS1-IO/ade6-L469 ura4-D18 his3-D1 leu1-32 arg3-D4* | This study |
| MCW7213 | *h^+^ pcf2*∆*::natMX4 ade6-M375 int*::*pUC8/his3^+^/RTS1-AO/ade6-L469 ura4-D18 his3-D1 leu1-32 arg3-D4* | This study |
| MCW8359 | *h^+^ pcf2*∆*::natMX4 ade6*∆::*RTS1-AO-hphMX4* (12.4 kb from *ade6*)int::*ade6-M375 int*::*pUC8/his3^+^/ade6-L469/kanMX6 ura4-D18 his3-D1 leu1-32 arg3-D4* | This study |
| MCW7147 | *h^+^ pcf3*∆*::LEU2 ade6-M375 int*::*pUC8/his3^+^/RTS1-IO/ade6-L469 ura4-D18 his3-D1 leu1-32 arg3-D4* | This study |
| MCW7149 | *h^+^ pcf3*∆*::LEU2 ade6-M375 int*::*pUC8/his3^+^/RTS1-AO/ade6-L469 ura4-D18 his3-D1 leu1-32 arg3-D4* | This study |
| MCW8360 | *h^+^ pcf3*∆*::LEU2 ade6*∆::*RTS1-AO-hphMX4* (12.4 kb from *ade6*)int::*ade6-M375 int*::*pUC8/his3^+^/ade6-L469/kanMX6 ura4-D18 his3-D1 leu1-32 arg3-D4* | This study |
