# Supplementary material for: Factors affecting template switch recombination associated with restarted DNA replication
Source: eLife. 2019 Jan 22;8:e41697. doi: 10.7554/eLife.41697 (PMC6358216; doi:10.7554/eLife.41697)
Supplement: Supplementary file 3. [file elife-41697-supp3.docx]

**SUPPLEMENTARY FILE 3**

Oligonucleotides

| Oligonucleotide | Nucleotide sequence (5´ to 3´) |
| --- | --- |
| oMW706 | **AAAGGCCTCGCTTCTCGAG** |
| oMW707 | **AGCAGCATACGCTAAAATC** |
| oMW1621 | **TATCAGCTGAGGCACGCTAAGGTGATGAG** |
| oMW1622 | **AATGTCGACTTCAACTGAACCTCGTCGTC** |
| oMW1623 | **TATGAGCTCGTAGTATCAAAGTGTAGTG** |
| oMW1624 | **AATACTAGTCATTACCAAGCGACAATAGAG** |
| oMW1659 | **TATCAGCTGTATCCTTCTGCGTTAAT** |
| oMW1660 | **AATGTCGACGACGATTTCAAAAATAGTAT** |
| oMW1661 | **TATGAGCTCGCGACTCTTAATTTTCTCTCT** |
| oMW1662 | **AATACTAGTGAAATACATGAAATCACAACC** |
| oMW1663 | **TATCAGCTGTTTTTAAGGAGTTCAAGACTT** |
| oMW1664 | **AATGTCGACTACAGCAGCAGTAAACC** |
| oMW1665 | **TATGAGCTCACAATTCTTCTGATATATTAA** |
| oMW1666 | **AATACTAGTTCCAGAGTTGAATGTTT** |
